# Dietary rapamycin supplementation reverses age‐related vascular dysfunction and oxidative stress, while modulating nutrient‐sensing, cell cycle, and senescence pathways

**DOI:** 10.1111/acel.12524

**Published:** 2016-09-22

**Authors:** Lisa A. Lesniewski, Douglas R. Seals, Ashley E. Walker, Grant D. Henson, Mark W. Blimline, Daniel W. Trott, Gary C. Bosshardt, Thomas J. LaRocca, Brooke R. Lawson, Melanie C. Zigler, Anthony J. Donato

**Affiliations:** ^1^ Division of GeriatricsDepartment of Internal MedicineSalt Lake CityUTUSA; ^2^ Veteran's Affairs Medical Center‐Salt Lake CityGeriatrics Research Education and Clinical CenterSalt Lake CityUTUSA; ^3^ Department of Exercise and Sports ScienceUniversity of UtahSalt Lake CityUTUSA; ^4^ Department of Integrative PhysiologyUniversity of Colorado BoulderBoulderCOUSA; ^5^ Department of BiochemistryUniversity of UtahSalt Lake CityUTUSA

**Keywords:** aging, AMPK, arterial stiffness, endothelial function, mTOR, oxidative stress, rapamycin

## Abstract

Inhibition of mammalian target of rapamycin, mTOR, extends lifespan and reduces age‐related disease. It is not known what role mTOR plays in the arterial aging phenotype or if mTOR inhibition by dietary rapamycin ameliorates age‐related arterial dysfunction. To explore this, young (3.8 ± 0.6 months) and old (30.3 ± 0.2 months) male B6D2F1 mice were fed a rapamycin supplemented or control diet for 6–8 weeks. Although there were few other notable changes in animal characteristics after rapamycin treatment, we found that glucose tolerance improved in old mice, but was impaired in young mice, after rapamycin supplementation (both *P* < 0.05). Aging increased mTOR activation in arteries evidenced by elevated S6K phosphorylation (*P* < 0.01), and this was reversed after rapamycin treatment in old mice (*P* < 0.05). Aging was also associated with impaired endothelium‐dependent dilation (EDD) in the carotid artery (*P* < 0.05). Rapamycin improved EDD in old mice (*P* < 0.05). Superoxide production and NADPH oxidase expression were higher in arteries from old compared to young mice (*P* < 0.05), and rapamycin normalized these (*P* < 0.05) to levels not different from young mice. Scavenging superoxide improved carotid artery EDD in untreated (*P* < 0.05), but not rapamycin‐treated, old mice. While aging increased large artery stiffness evidenced by increased aortic pulse‐wave velocity (PWV) (*P* < 0.01), rapamycin treatment reduced aortic PWV (*P* < 0.05) and collagen content (*P* < 0.05) in old mice. Aortic adenosine monophosphate‐activated protein kinase (AMPK) phosphorylation and expression of the cell cycle‐related proteins PTEN and p27kip were increased with rapamycin treatment in old mice (all *P* < 0.05). Lastly, aging resulted in augmentation of the arterial senescence marker, p19 (*P* < 0.05), and this was ameliorated by rapamycin treatment (*P* < 0.05). These results demonstrate beneficial effects of rapamycin treatment on arterial function in old mice and suggest these improvements are associated with reduced oxidative stress, AMPK activation and increased expression of proteins involved in the control of the cell cycle.

## Introduction

Aging is a primary risk factor for cardiovascular diseases (CVDs) (D'Agostino *et al*., 2008), the leading cause of death in the USA (Mozaffarian *et al*., 2015). CVDs are primarily diseases of the arteries and are associated with reduced endothelial function and augmented large artery stiffening (Yeboah *et al*., 2007; Mitchell *et al*., 2010). It is well established that both of these characteristics are observed even in healthy older adults (Mitchell *et al*., 2004; Seals *et al*., 2011) and that they are independent predictors of future CVD diagnosis (Yeboah *et al*., 2007; Rossi *et al*., 2008). Although the initiating events of arterial aging are still unknown, several critical factors have been established, including reduced nitric oxide (NO) bioavailability, increased arterial oxidative stress, and increased arterial fibrosis.

Oxidative stress, marked by excess superoxide, is known to be an important contributor to age‐associated arterial dysfunction as a result of the scavenging of NO by superoxide. This results in blunting of endothelial‐mediated vasodilation and augmentation of large artery stiffness (Donato *et al*., 2013). There is a shift in redox balance in arteries and endothelial cells from old mice and humans that results from enhanced superoxide production, due to augmented activity of cytosolic enzymes such as NADPH oxidases (NOX), and inadequate upregulation of cellular antioxidant defenses (Donato *et al*., 2007, 2013). Excessive superoxide‐associated oxidative stress leads to stiffening of large elastic arteries (Fleenor *et al*., 2014) with aging by producing changes in the major structural proteins (collagen and elastin) (Henson *et al*., 2014). This fibrotic state is exacerbated by age‐related glucose intolerance contributing to postprandial hyperglycemia, which increases endothelial oxidative stress and enhances advanced glycation end product‐associated cross‐linking of collagen (Avendano *et al*., 1999; Goldin *et al*., 2006). Collectively, these mechanisms play an important role in age‐related arterial dysfunction.

Nutrient‐sensing molecular pathways, such as adenosine monophosphate‐activated protein kinase (AMPK), sirtuins, and mammalian target of rapamycin (mTOR) are closely related to overall metabolic function (Howell & Manning, 2011; Mihaylova & Shaw, 2011) and are dysregulated with advancing age (Lesniewski *et al*., 2012; Donato *et al*., 2013). These pathways have been implicated in the lifespan extending effects of caloric restriction (CR) (Greer *et al*., 2007; Medvedik *et al*., 2007), as well as in the arterial aging phenotype (Lesniewski *et al*., 2012; Donato *et al*., 2013). Although studies examining the direct effects of AMPK and sirtuin‐1 activation have revealed selective beneficial effects on arterial endothelial function in aged mice (Lesniewski *et al*., 2012; Gano *et al*., 2014), less is known about the effects of mTOR inhibition on age‐associated arterial dysfunction.

Importantly, inhibition of mTOR by dietary rapamycin has been recently demonstrated to delay age‐associated diseases and extend lifespan in mice (Harrison *et al*., 2009; Wilkinson *et al*., 2012). Moreover, lifespan extension was also seen when dietary treatment was started in middle age (Harrison *et al*., 2009). While the mechanisms underlying the beneficial effects of rapamycin treatment and mTOR inhibition are incompletely understood, it is clear that signaling through the mTOR pathway modulates numerous cell cycle proteins, which may have beneficial effects in a variety of tissues. Furthermore, in conditions such as diabetic kidney disease, mTOR inhibition has been demonstrated to decrease NOX4 expression and reduce superoxide/reactive oxygen species (ROS) generation (Eid *et al*., 2013). Taken together, these studies suggest that dietary rapamycin has substantial promise to counteract mechanisms responsible for arterial aging. However, the impact of mTOR inhibition by dietary rapamycin on arterial function in aged animals remains unknown.

In this study, we used an established mouse model of arterial aging to assess the role of mTOR activity in endothelial dysfunction and large artery stiffening with advancing age. We hypothesized that age‐associated increases in arterial mTOR activation would be associated with impairments in vascular function (i.e. reduced endothelium‐dependent dilation and increased large artery stiffness), increased oxidative stress and reduced NO, increased cellular oxidant enzymes, decreased AMPK activation, and increased extracellular matrix fibrosis in the large arteries. To investigate the role of mTOR activity further, we hypothesized that dietary inhibition of mTOR with rapamycin in old mice would ameliorate this arterial aging phenotype.

## Results

### Animal characteristics

Body mass, tissue mass, and mean arterial pressures for young and old untreated and rapamycin‐treated mice can be found in Table 1 (*N* = 6–15/group). Because rapamycin is clinically used as an immunosuppressant, we assessed the complete blood count (CBC) and differential counts in peripheral blood collected at sacrifice from old untreated and rapamycin‐treated mice. We found that rapamycin did not impact total white blood cell, differential, hemoglobin, hematocrit, or platelet counts (Table S1, Supporting information). Spleen mass, measured at sacrifice, was reduced with advancing age and after rapamycin treatment in young mice, but increased after rapamycin in old mice (Table 1). To determine whether these changes in mass were the result of tissue congestion, the dry weight of the spleen was calculated and expressed as a percent of tissue wet weight. Percent of dry weight of the spleen was reduced by aging (young: 14.3 ± 1.2% vs. old: 10.9 ± 0.9%, *P* < 0.05) and rapamycin treatment in young mice (young rap: 11.0 ± 0.8%, *P* < 0.05), suggesting tissue congestion in these groups. However, rapamycin increased dry weight of the spleens from old mice (old rap: 14.0 ± 0.9%, *P* < 0.05 vs. old untreated) and may be indicative of reduced congestion or increased cellularity in this tissue.

**Table 1:** Age, body and tissue mass, blood pressure and blood glucose, plasma insulin, and homeostatic model assessment of beta‐cell function (HOMA‐B) and insulin resistance (HOMA‐IR) in untreated and rapamycin‐treated young and old mice

	Young	Old	Young Rap	Old Rap	*N*'s
Age (months)	3.8 ± 0.6	30.3 ± 0.2^a^	4.0 ± 0.0	30.3 ± 0.2^a^	9–15
Body mass (g)	32.5 ± 1.6	35.6 ± 1.4	29.3 ± 0.4	36.9 ± 1.3^a^	8–15
Gastrocnemius mass (mg)	243 ± 23	150 ± 8^a^	170 ± 2^a^	139 ± 5^a^	7–12
Heart mass (mg)	157 ± 10	202 ± 7^a^	134 ± 4^a^	209 ± 6^a^	7–12
Liver mass (g)	1.61 ± 0.06	1.97 ± 0.07^a^	1.21 ± 0.04^a^	1.87 ± 0.08^a^	7–12
Spleen mass (mg)	82 ± 5	75 ± 13^a^	64 ± 2^a^	116 ± 21^a^^,^ b	6–8
Epididymal WAT mass (mg)	679 ± 99	327 ± 73	456 ± 23	576 ± 102	7–12
Mean arterial pressure (mmHg)	109 ± 3	101 ± 5	101 ± 4	95 ± 3	7–8
AUC_glc_ (%fasted)	11210 ± 365	12410 ± 388^a^	14620 ± 1127^a^	11241 ± 333^b^	8–12
Fed blood glucose (mg dL^−1^)	142 ± 3	125 ± 6^a^	134 ± 3	115 ± 5^a^	8–13
Fed plasma insulin (μU mL^−1^)	23.8 ± 4.3	47.3 ± 7.7^a^	30.8 ± 2.9	39.0 ± 5.1	7–10
HOMA‐IR (%)	4.4 ± 0.5	5.5 ± 0.6	4.2 ± 0.4	5.1 ± 0.7	7–10
HOMA‐B (%)	110.8 ± 10.9	175.5 ± 20.4^a^	117.5 ± 8.4	180.6 ± 7.9^a^	7–10

Rap, rapamycin treated, WAT, white adipose tissue.

*N* = 6–15/group.

Data are means ± SEM, *P* < 0.05.

a Denotes difference from young.

b Denotes difference from untreated old.

Glucose tolerance was impaired in old untreated compared to young untreated mice (*P* < 0.05), evidenced by a greater area under the curve for glucose (AUC_glc_) during the GTT (Table 1). Rapamycin treatment improved glucose tolerance, that is resulted in a reduction in the AUC_glc_ (*P* < 0.01) in old mice (Table 1). In contrast, rapamycin impaired glucose tolerance in young mice such that there was an increased AUC_glc_ (*P* < 0.05) during the GTT. Blood glucose was lower, and plasma insulin was higher in the old compared to young untreated mice (Table 1). Although HOMA‐IR% was not different, HOMA‐B% was higher in old compared to young untreated mice (Table 1). However, blood glucose, plasma insulin, HOMA‐IR%, and HOMA‐B% were not impacted by rapamycin treatment in the young or old mice (Table 1). GTT responses are provided in Fig. S1 (Supporting information).

### Enhanced arterial mTOR activation with aging is reversed after rapamycin treatment

**Figure 1:**
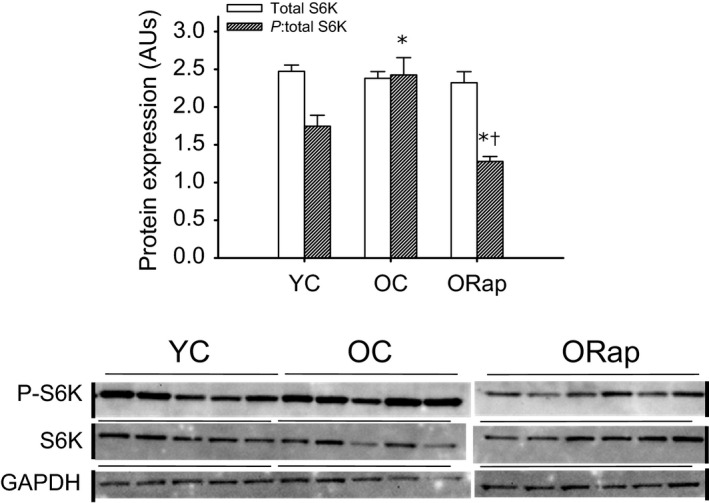
Expression and activation of the mammalian target of rapamycin (mTOR) target, ribosomal S6 kinase (S6K) in aortas of young and old untreated and old rapamycin‐treated mice. Total (open bars) and the ratio of phosphorylated (P‐) to total (hashed bars) S6K, in aortas excised from young and old untreated and rapamycin‐treated mice (*N* = 5–6/group). Blot is shown next to summary data. Black vertical lines indicate where blot image was digitally cut to reposition groups in the same order as the summary graphs. * denotes difference from Young, and † denotes difference from old. Differences were assessed by one‐way ANOVA with LSD post hoc. Data are means ± SEM,* P* ≤ 0.05

Although total protein expression of mTOR target S6K, assessed by Western blotting, did not differ with aging or rapamycin treatment, phosphorylation of S6K, a marker of activation, was higher in aorta of old compared to young mice (*P* < 0.01) (Fig. 1). Dietary rapamycin treatment reduced S6K activation relative to both young (*P* < 0.05) and old untreated (*P* < 0.01) mice (Fig. 1).

### mTOR inhibition in old mice by dietary rapamycin ameliorates oxidative stress‐mediated endothelial dysfunction

**Figure 2:**
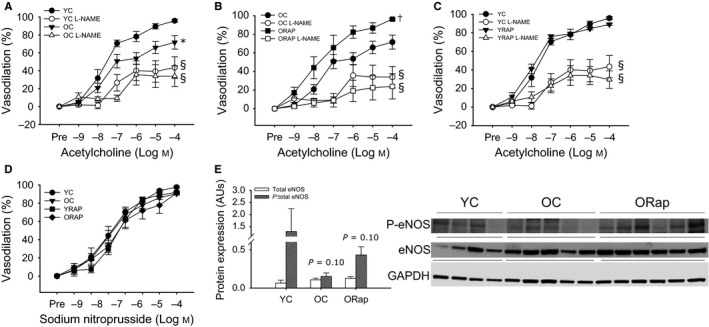
Endothelium‐dependent dilation in young and old untreated and old rapamycin‐treated mice. (A) Dose responses to acetylcholine in the absence or presence of the nitric oxide synthase inhibitor, L‐NAME, in carotid arteries from young (YC) (*N* = 6) and old (OC) (*N* = 9) untreated mice. (B) Dose responses to acetylcholine in the absence or presence of L‐NAME in carotid arteries from OC (*N* = 9) and old rapamycin (ORAP) (*N* = 9)‐treated mice. (C) Dose responses to acetylcholine in the absence or presence L‐NAME in carotid arteries from YC (*N* = 9) and young rapamycin (YRAP) (*N* = 9)‐treated mice. (D) Dose responses to the endothelium‐independent vasodilator, sodium nitroprusside, in carotid arteries from YC, OC, YRAP, and ORAP mice (*N* = 4–8/group). * denotes group difference from YC, † denotes group differences from OC, and § denotes difference with L‐NAME from ACh alone. Differences in dose responses were assessed by repeated‐measures ANOVA. (E) Total (open bars) and the ratio of ser1177 phosphorylated (P‐) to total (hashed bars) expression of endothelial nitric oxide synthase (eNOS) in aortas excised from young and old untreated and rapamycin‐treated mice (*N* = 4–6/group), *P* = 0.1 denotes tendency for difference in the ratio for young vs. old and old vs. old Rap. Blot images are provided next to summary data. Differences were assessed by one‐way ANOVA. Data are means ± SEM,* P* ≤ 0.05

To determine the impact of aging and rapamycin treatment on endothelial function and NO bioavailability, carotid arteries were excised and cannulated in the stage of an inverted microscope and dose responses to acetylcholine (ACh), an endothelium‐dependent dilator (EDD), and sodium nitroprusside, an endothelium‐independent vasodilator, were performed. EDD to ACh was reduced in carotid arteries of old compared to young mice (*P* < 0.05). This was the result of reduced NO bioavailability as evidenced by a loss of the age‐associated differences in dilation after nitric oxide synthase inhibition by L‐NAME (Fig. 2A). Dietary rapamycin treatment improved EDD (*P* < 0.01) and NO bioavailability in old mice compared to untreated old mice and restored these measurements to those of young controls (Fig. 2B). Rapamycin treatment was without effect on EDD or NO bioavailability in young mice (Fig. 2C). There were no differences in endothelium‐independent dilation to sodium nitroprusside (SNP) with aging or rapamycin treatment in either young or old mice (Fig. 2D). There were no differences in total aortic eNOS protein expression with aging or rapamycin treatment in old mice (Fig. 2E). Still, aging tended to decrease (*P* = 0.1) and rapamycin tended to increase (*P* = 0.1 vs. old) phosphorylation of eNOS at the activating site, ser1177, in old mice (Fig. 2E).

**Figure 3:**
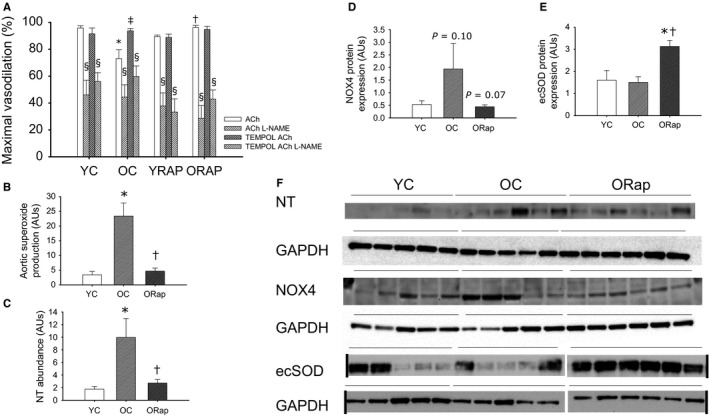
Oxidative stress in arteries of young and old untreated and rapamycin‐treated mice. (A) Maximal vasodilation to acetylcholine (ACh) in the absence or presence of the nitric oxide synthase inhibitor, L‐NAME, and the superoxide scavenger, TEMPOL, in carotid arteries from young untreated (*N* = 6), young rapamycin (Rap)‐treated (*N* = 6), old (OC) (*N* = 6) untreated and old Rap‐treated mice (*N* = 6). * denotes difference from young ACh alone, †denotes difference from Old ACh alone, ‡ denotes difference after TEMPOL from old ACh alone, and § denotes difference with L‐NAME from paired ACh response. Differences were assessed by repeated‐measures ANOVA with LSD post hoc. (B) Superoxide production measured by electron paramagnetic resonance in aortas from young and old untreated and old Rap‐treated mice (*N* = 6–9/group). (C) Abundance of nitrotyrosine (NT), a marker of oxidative stress, in aortas from young and old untreated and old Rap mice (*N* = 5–6/group). (D) NADPH oxidase 4 (NOX4) expression in aortas from young and old untreated and old Rap mice (*N* = 5‐6/group). *P* = 0.1 vs. young, *P* = 0.07 vs. old. (E) Extracellular superoxide dismutase (ecSOD) expression in aortas from young and old untreated and old Rap mice (*N* = 5–6/group). (F) Blot images for NT, NOX4, ecSOD, and the associated normalizer, GAPDH. Black vertical lines indicate where blot image was digitally cut to reposition groups in the same order as the summary graphs, where applicable. Expression is presented as a ratio to GAPDH to account for differences in protein loading. Differences were assessed by one‐way ANOVA with LSD post hoc. For B–E, * denotes difference from young untreated and † denotes difference after old untreated. Data are means ± SEM,* P* ≤ 0.05

Maximal dilation to ACh was reduced with aging (*P* < 0.05) (Fig. 3A). Both dietary rapamycin and *in vitro* treatment with the superoxide dismutase mimetic, TEMPOL, restored maximal dilation in old mice (both *P* ≤ 0.05) (Fig. 3A). TEMPOL treatment of carotid arteries from young untreated and rapamycin‐treated mice was without effect on maximal dilation (Fig. 3A). In contrast to untreated old mice, *in vitro* treatment of carotid arteries with TEMPOL did not improve maximal dilation in arteries from old rapamycin‐treated mice (Fig. 3A). These findings indicate that increased oxidative stress underlies the observed reductions in EDD with aging and suggest that rapamycin improves EDD by reducing oxidative stress.

L‐NAME treatment of isolated arteries removed all group and treatment differences (Fig. 3A), indicating that the impairments with aging and improvements in dilation after rapamycin or TEMPOL in old mice result from alterations in NO bioavailability downstream of increased oxidative stress. Superoxide production, measured by electron paramagnetic resonance (EPR) (Fig. 3B), and nitrotyrosine abundance (Fig. 3C), markers of oxidative stress, were increased in the aorta with aging (*P* < 0.05), and this was reversed after dietary rapamycin treatment (*P* < 0.05). The protein expression of the oxidant enzyme NOX4 tended to be increased with aging (*P* = 0.10) and reduced after rapamycin (*P* = 0.07) (Fig. 3D). Likewise, gene expression for another NADPH oxidase isoform, NOX2, was increased with advancing age (1.0 ± 0.4 vs. 12.1 ± 8.1 AU, *P* < 0.05) and reduced after rapamycin treatment in aortas from old (0.5 ± 0.2 AU, *P* < 0.05 vs. old), but not young (1.7 ± 0.4 AU) mice. Although not different with aging, the expression of the antioxidant extracellular superoxide dismutase (ecSOD) was increased in the aortas of old mice after dietary rapamycin compared to both young and old untreated mice (both *P* < 0.01) (Fig. 3E).

### Dietary rapamycin reduces large elastic artery stiffness in old mice and selectively modifies arterial wall composition

**Figure 4:**
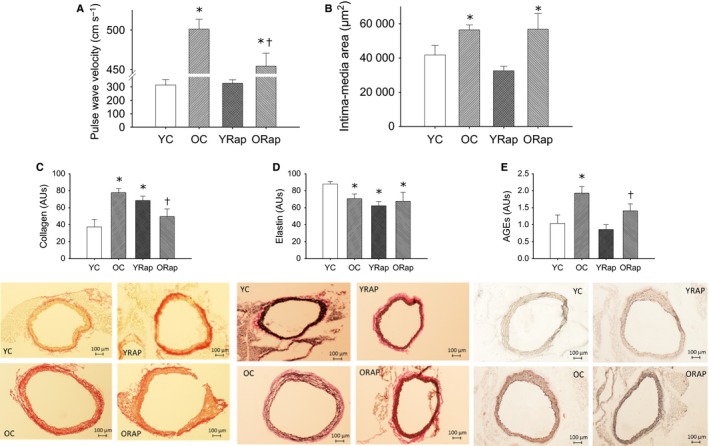
Large artery stiffness and arterial morphology and composition in young and old untreated and rapamycin‐treated mice. (A) Aortic stiffness, assessed by pulse‐wave velocity, in young (Y) and old (O) untreated and rapamycin (Rap)‐treated mice, (B) intima‐media area visualized by Maisson trichrome, (C) collagen expression measured by picrosirius red staining, (D) elastin expression measured by Verhoff Van Geisson staining, and (E) advanced glycation endproducts (AGEs) quantified by immunohistochemistry on histological sections of aortas from young and old untreated and rapamycin‐treated mice. Representative images are provided below the summary data. Differences were assessed by one‐way ANOVA with LSD post hoc.* denotes difference from untreated young, and † denotes difference from untreated old. Data are means ± SEM,* P* ≤ 0.05

Large elastic artery stiffness, assessed by *in vivo* aortic pulse‐wave velocity (PWV), was increased with aging (*P* < 0.001) and was improved (*P* < 0.05 vs. old), but not restored to young values (*P* < 0.01 vs. young), in old mice after rapamycin treatment (Fig. 4A). Rapamycin treatment was without effect on PWV in young mice (Fig. 4A). The intima‐media area of the aorta was increased in old mice (*P* < 0.05), but rapamycin treatment had no effect on intima‐media area in young or old mice (Fig. 4B).

To determine whether the structural changes underlie changes in large artery stiffness with aging and rapamycin, aortic collagen (assessed by pricrosirius red stain), elastin (assessed by Verhoff's Van Geison stain, advanced glycation end products (AGEs, assessed by immunohistochemistry), and calcification (assessed by Von Kossa staining) were assessed on histological aortic sections. With aging in the aorta, there was increased collagen (*P* < 0.01) (Fig. 4C), decreased elastin (*P* < 0.01) (Fig. 4D), and increased abundance of AGEs (*P* < 0.01) (Fig. 4E). There was no evidence of calcification of the aorta with aging (data not shown).

In old mice, dietary rapamycin reduced aortic collagen (*P* < 0.05) (Fig. 4C), but was without effect on elastin content (Fig. 4D). In contrast, in young rapamycin‐treated mice, collagen was higher and elastin was lower in the aorta compared to untreated young mice (Fig. 4C,D), despite there being no differences in PWV. AGEs were reduced (*P* < 0.05) in aortas of old, but not young mice after dietary rapamycin treatment (Fig. 4E).

### mTOR inhibition increases AMPK activation and cell cycle regulatory proteins and decreases a marker of senescence in aorta of old mice

**Figure 5:**
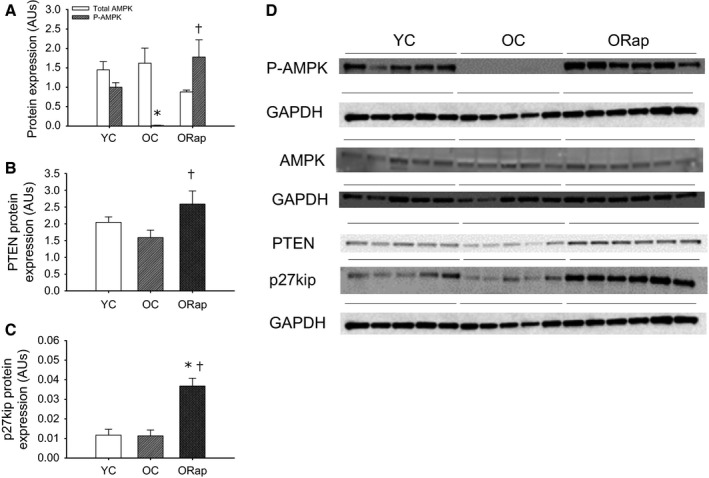
Expression and activation of adenosine monophosphate‐activated protein kinase (AMPK) and cell cycle‐related proteins in aortas of young and old untreated and old rapamycin‐treated mice. (A) Total (open bars) and the ratio of phosphorylated (P‐) to total (hashed bars) AMPK in aortas excised from young and old untreated and rapamycin‐treated mice (*N* = 5–6/group). Expression of (B) phosphatase and tensin homolog, PTEN, and (C) the cyclin‐dependent kinase inhibitor, p27kip, in aortas excised from young and old untreated and rapamycin‐treated mice (*N* = 5–6/group). Expression was normalized to GAPDH and blot images are provided in panel (D). * denotes difference from young, and † denotes difference from old. Differences were assessed by one‐way ANOVA with LSD post hoc. Data are means ± SEM,* P* ≤ 0.05.

Phosphorylation, but not total protein expression, of AMPK was lower in aorta of old compared to young mice (*P* < 0.05, Fig. 5). Dietary rapamycin treatment in old mice did not impact total AMPK protein expression, but increased phosphorylation of AMPK (*P* < 0.01) compared to untreated old mice (Fig. 5A). Although not different with aging, the cell cycle regulatory proteins, PTEN (*P* < 0.05 old Rap vs. old) (Fig. 5B) and p27kip (*P* < 0.01 old Rap vs. young and old) (Fig. 5C) were increased in the aortas of old mice after dietary rapamycin treatment. Gene expression, assessed by quantitative PCR, for the senescence‐related cyclin‐dependent kinase inhibitor, p19, was increased with advancing age (young: 1.0 ± 0.3 vs. Old: 8.0 ± 3.2 AU, *P* < 0.05). Rapamycin was without effect on p19 gene expression in aortas from young mice (1.1 ± 0.3 AU), but decreased p19 in aortas of old mice (1.6 ± 0.7 AU, *P* < 0.05 vs. old).

## Discussion

The novel findings of the present study are that (a) compared with young mice, mTOR activation was increased in the large arteries of old mice and was associated with vascular oxidative stress, impaired endothelial function, increased large elastic artery stiffness, decreased AMPK activation, and increased senescence marker p19, but no change in the expression of cell cycle‐related proteins, PTEN or p27kip, and (b) dietary rapamycin treatment of old mice reversed the arterial aging phenotype and was associated with decreased oxidative stress, increased arterial AMPK activation, increased expression of PTEN and p27kip, and decreased senescence marker p19. We also provide evidence that although rapamycin can lead to reductions in glucose tolerance in young mice as reported previously by others (Lamming *et al*., 2012); however, old mice demonstrate improved glucose tolerance after rapamycin treatment. Our results demonstrate that enhanced mTOR signaling is an important factor in age‐associated vascular dysfunction and may also play a role in glucose intolerance with aging. These findings also suggest that changes in the cell cycle regulation/cell fate may underlie at least part of the beneficial vascular effects of rapamycin, a possibility requiring further elucidation.

### Rapamycin and endothelial function

Aging is associated with endothelial dysfunction and reduced NO bioavailability. Here, we find that activation, indicated by ser1177 phosphorylation of eNOS, tended to be reduced with aging and improved after rapamycin. Although such changes in eNOS activation may contribute to the age‐related impairments and rapamycin‐related improvements in EDD and NO bioavailability, they cannot fully explain these effects. Age‐associated endothelial dysfunction is characterized by an oxidative stress‐mediated reduction in NO bioavailability. Similar to what we have previously demonstrated after both short‐term (Rippe *et al*., 2010) and lifelong CR (Donato *et al*., 2013), as well as after chronic aerobic exercise in old mice (Durrant *et al*., 2009), rapamycin treatment decreased arterial oxidative stress, evidenced by decreased nitrotyrosine content, a cellular marker of oxidative stress, and reduced superoxide production measured by EPR. Age‐associated arterial oxidative stress results from an increase in oxidant production, at least in part as a consequence of increased expression and activity of NADPH oxidase, in the absence of adequate compensatory antioxidant increases (Durrant *et al*., 2009; Rippe *et al*., 2010; Donato *et al*., 2013). Here, we extend these earlier findings to demonstrate that both the NOX4 and NOX2 isoforms of NADPH oxidase are increased in arteries of old mice and that mTOR inhibition is associated with reduced expression of both of these NOX isoforms in old mice. This is similar to changes reported for isoforms of NADPH oxidase after CR (Rippe *et al*., 2010; Donato *et al*., 2013). We have also demonstrated that the vascular protection afforded by CR is associated with an increased expression of a critical antioxidant, ecSOD. Here, we find a similar increase in ecSOD expression after rapamycin treatment in old mice. As ecSOD is a critical factor in the protection of NO in its diffusion from endothelial cells through the extracellular space into vascular smooth muscle, increased ecSOD may explain, in part, the improvements in NO bioavailability after rapamycin treatment. However, unlike CR (Rippe *et al*., 2010; Donato *et al*., 2013) or chronic aerobic exercise (Durrant *et al*., 2009), the expression of other SOD isoforms, such as Mn or CuZn, was not impacted by rapamycin treatment (data not shown). Thus, rapamycin treatment in old mice appears to mimic selective beneficial effects of both CR and exercise.

### Rapamycin and large elastic artery stiffness

The effects of rapamycin treatment on large elastic artery stiffness in old mice largely mimic those of lifelong CR. Lifelong CR reduced aortic PWV compared to *ad libitum* fed age‐matched mice (Donato *et al*., 2013), and this was associated with reduced area of the medial wall, decreased collagen and increased elastin content of the aorta (Donato *et al*., 2013). Here, we find that dietary rapamycin improves, but does not completely normalize, aortic stiffness in old mice. This reduction in arterial stiffness was associated with a rapamycin‐induced decrease in collagen and AGEs in the aorta in the absence of changes in intima‐media thickness or aortic elastin content. The findings of the present study are consistent with a ‘CR mimetic’ effect of rapamycin on age‐associated large elastic artery stiffening, and an incomplete phenocopy may be due to the shorter length of treatment compared to lifelong CR. Furthermore, the reduction in AGEs observed in the present study suggests that the beneficial effects of rapamycin on large elastic artery stiffness may be mediated, at least in part, by a reduction in the cross‐linking of collagens by AGEs, an effect that may be related to the improved glucose metabolism after rapamycin treatment in old mice (Beisswenger *et al*., 1993).

### Rapamycin and AMPK activation

AMPK activation, in response to low cellular energy status, leads to a reduction in mTOR signaling via inhibition of S6K (Gwinn *et al*., 2008). Likewise, inhibition of mTOR after rapamycin treatment of MCF‐7 breast cancer cells leads to increased AMPK activation, indicating a reciprocal relation between these energy‐sensing pathways (Zakikhani *et al*., 2010). In addition to effects on metabolism, AMPK activation increases eNOS activation in cultured endothelial cells, suggestive of a vasoprotective effect (Murakami *et al*., 2006). Our laboratory has previously demonstrated that pharmacological activation of AMPK, via *in vivo* administration of AICAR, reverses superoxide‐mediated suppression of endothelium‐dependent dilation in the carotid arteries of old mice, although this effect did not result from an increase in NO bioavailability, but rather from increased reliance on another endothelial vasodilator, endothelium‐derived hyperpolarizing factor (EDHF) (Lesniewski *et al*., 2012). Here, we demonstrate the cross talk between mTOR inhibition and AMPK activation in arteries, with rapamycin treatment of old mice leading to increased arterial AMPK activation. However, unlike direct pharmacological activation of AMPK by AICAR, increased vasodilation in old mice after rapamycin resulted from increased NO bioavailability. Still, the beneficial effects of both rapamycin and AICAR on arterial endothelial vasodilation appear to be predominately mediated through a reduction in the suppression of vasodilation by oxidative stress. This effect to reduce oxidative stress and improve vasodilation via either AICAR or rapamycin occurs regardless of the endogenous vasodilator (i.e. NO or EDHF) being impacted.

### Rapamycin, cell fate, and senescence

Advancing age is associated with cellular senescence (Herbig *et al*., 2006). Here, we demonstrate that although aging did not impact the expression of the tumor suppressor, PTEN, or the cell cycle inhibitor, p27kip, their expressions were increased after rapamycin treatment. PTEN is a negative regulator of protein kinase B (AKt) signaling with antiproliferative effects and a reduction in PTEN‐mediated inhibition of Akt in old mice may underlie the reduction in p27kip (Sun *et al*., 2014). p27kip is a member of the Cip/kip family of cyclin‐dependent kinase inhibitors that act to block progression through the G_1_/S transition of the cell cycle (Pestell *et al*., 1999). In addition, we found that rapamycin reversed the age‐associated increase in p19, a cyclin‐dependent kinase inhibitor that is associated with cellular senescence. Taken together, these results indicate that rapamycin treatment in old mice may reduce growth signaling in arteries and perhaps favor the clearing of dysfunctional senescent cells/tissue, which could in part explain the improvements in arterial vasodilation and stiffness we observed.

### Differential effects of rapamycin on glucose/insulin metabolism in young and old

Although not a primary outcome of our study, our results are consistent with those of previous investigations (Lamming *et al*., 2012; Fang *et al*., 2013), indicating that short‐term rapamycin treatment in young mice leads to glucose and insulin intolerance. In contrast, long(er) term treatment with rapamycin, from 20 weeks up to 2 years, improved metabolic function and reduced adiposity in mice (Harrison *et al*., 2009; Anisimov *et al*., 2011; Fang *et al*., 2013). Here, we find that short‐term rapamycin treatment of old mice improves glucose tolerance. In young mice, rapamycin treatment increased collagen and decreased elastin in the aortas, and although such differences should act to increase large elastic artery stiffness, these structural changes were not sufficient to change aortic PWV. When applied to our young mice, the findings of Fang *et al*. (2013) suggest that the deleterious effects of short‐term rapamycin treatment on glucose‐insulin metabolism would resolve if treatment were sustained. However, it is not known if a longer period of treatment in young mice might also lead to potentially deleterious changes in arterial structure or function. Although earlier reports demonstrating lifespan extending and beneficial metabolic effects of long‐term rapamycin treatment (initiated in middle age) (Harrison *et al*., 2009; Miller *et al*., 2014) argue against a deleterious effect of prolonged treatment, no information is available on other biomarkers, including vascular function.

### Rapamycin/mTOR inhibition: a potential CR mimetic?

Treatment of mice with rapamycin beginning in early or middle age can extend lifespan and reduce many age‐related phenotypes including liver degeneration, myocardial nuclear abnormalities, endometrial cystic hyperplasia, adrenal tumors, tendon elasticity, age‐related loss of spontaneous activity, cognitive deficits, age‐related cardiac dysfunction, altered blood cell count, cellular senescence, self‐renewal and activity of hematopoietic stem cells, and turnover of hepatic proteins (Chen *et al*., 2009; Harrison *et al*., 2009; Miller *et al*., 2011, 2014; Majumder *et al*., 2012; Wilkinson *et al*., 2012; Flynn *et al*., 2013; Neff *et al*., 2013; Karunadharma *et al*., 2015). These lifespan extending and anti‐aging effects of rapamycin are similar to those previously reported in mice undergoing lifelong CR (reductions of 40% of caloric intake). Previous work by our laboratories has demonstrated that CR, either lifelong or short‐term in old mice, can ameliorate age‐related arterial dysfunction (Rippe *et al*., 2010; Donato *et al*., 2013), and these changes are associated with blunted mTOR signaling in old arteries (Donato *et al*., 2013). These findings further support the role mTOR inhibition in the vasoprotective effects of both CR and dietary rapamycin in old mice. Unlike AMPK activation (AICAR) (Lesniewski *et al*., 2012) and SIRT‐1 activation (SRT1720) (Gano *et al*., 2014), these effects of rapamycin fully recapitulate preserved endothelial function and nitric oxide bioavailability observed with both lifelong and short‐term CR; therefore, rapamycin appears to be the most viable CR mimetic tested in our laboratory.

### Summary and future directions

Age‐associated vascular dysfunction is an important contributing factor to increased CVD risk in older adults. Our results indicate that increased mTOR activation may underlie vascular oxidative stress, endothelial dysfunction, increases in large elastic artery stiffness, and structural adaptations in the vessel wall with advancing age. Dietary rapamycin treatment reversed these deleterious arterial phenotypes and increased the expression of proteins involved in the control of the cell cycle. Our findings suggest that treatment with rapamycin or other ‘rapalogs’ holds promise for the treatment of arterial aging and, therefore, the potential prevention of age‐associated CVD. Future studies should explore the role of the individual mTOR complexes in age‐associated vascular dysfunction, as well as the impact of rapamycin on autophagy and cellular senescence.

## Experimental procedures

### Ethical approval

All animal procedures conformed to the Guide to the Care and Use of Laboratory Animals (NIH publication no. 85‐23, revised 2010) and were approved by the University of Colorado at Boulder, University of Utah and VAMC‐SLC Animal Care and Use Committees.

### Animals

Young male B6D2F1 mice were obtained from Charles River Inc, and old male mice were purchased from the aging colonies maintained at Charles River Inc. for the National Institute on Aging. All mice were housed in standard mouse cages in an animal care facility at the VAMC‐SLC or at the University of Colorado at Boulder on a 12‐h:12‐h light:dark cycle. Mice were fed either a custom control (Purina 5LG6/122 PPM Eudragit 3/8 Pellet, Test Diet) or rapamycin supplemented diet (14 mg kg^−1^ diet in Purina 56LG6/122 PPM Eudragit 3/8 Pellet, Test Diet) for 6 weeks, as described by Harrison *et al*. (2009). Assuming a daily food intake of approximately 5 g day^−1^ and an approximately 30‐g mouse, this yields a dose of 2.24 mg rapamycin kg^−1^ body weight day^−1^. Food and water were supplied *ad libitum*. Glucose tolerance was assessed by intraperitoneal glucose tolerance test (GTT) as previously described (Lesniewski *et al*., 2007; Donato *et al*., 2012). Briefly, in the morning after a 2‐h fast, blood glucose was collected from a tail nick (5 μl) and assessed with a Precision Xceed Pro Glucose Analyzer. Glucose was then administered (2 g glucose kg^−1^ body weight), and blood glucose was measured in whole blood at times 15, 30, 45, 60, and 90 min after injection. Area under the curve for glucose (AUC_glc_) during the GTT was calculated. Prior to tissue harvest, mice were euthanized via exsanguinations by cardiac puncture while under isoflurane anesthesia (Donato *et al*., 2009; Durrant *et al*., 2009). Whole blood from untreated and rapamycin‐treated old mice collected at sacrifice was used for total white blood cell and differential assessment. To do so, a standard veterinary differential was performed with 100 WBC counted and types identified thereby giving a percent for each type of WBC.

### *Ex vivo* arterial endothelium and vascular smooth muscle assessment

To assess endothelial function, carotid arteries were excised, cleared of surrounding tissue and cannulated in the stage of a pressure myograph (DMT Inc, Atlanta, GA, USA). Arteries were preconstricted with 2 μm phenylephrine, and endothelium‐dependent dilation and the NO contribution to dilation were measured in response to the cumulative addition of acetylcholine (1 × 10^−9^ to 1 × 10^−4^ mol L^−1^) in the absence or presence of the nitric oxide synthase inhibitor, L‐NAME (0.1 mmol L^−1^, 30 min), as described previously (Durrant *et al*., 2009). To assess superoxide‐mediated suppression of endothelial function, acetylcholine dose responses in the absence and presence of L‐NAME were performed in the contralateral carotid artery after incubation with the superoxide dismutase mimetic, TEMPOL (1 mmol/L, 1 h). Endothelium‐independent dilation was assessed in response to sodium nitroprusside (1 × 10^−10^ to 1 × 10^−4^ mol L^−1^) (Durrant *et al*., 2009). Vessel diameters were measured by MyoView software (DMT, Inc., Atlanta, GA, USA). All dose–response data are presented as percent of possible dilation after preconstriction to phenylephrine. Arteries failing to achieve ≥ 20% preconstriction were excluded. Sensitivity was defined as the concentration of ACh or SNP that elicited 50% of the maximal response (IC_50_). Sensitivities (IC_50_s) were calculated using BioDataFit 1.02. A regression was used to fit a sigmoidal model to individual dose responses yielding a dose for the half maximal response in log M units.

### Superoxide production

Production of superoxide was measured by electron paramagnetic resonance (EPR) spectrometry using the spin probe 1‐hydroxy‐3‐methoxycarbonyl‐2,2,5,5‐tetramethylpyrrolidine (CMH, Alexis Biochemicals). Stock solutions of CMH were prepared in ice‐cold deoxygenated Krebs–HEPES buffer (mmol L^−1^: NaCl, 99.01, KCl 4.69, CaCl_2_ 2.50, MgSO_4_ 1.20, K_2_HPO_4_ 1.03, NaHCO_3_ 25.0, glucose 11.10, and Na‐HEPES 20.00; pH 7.4) containing 0.1 mmol L^−1^ diethylenetriamine‐penta‐acetic acid and 5 μmol L^−1^ sodium diethyldithiocarbamate and pretreated with Chelex (Sigma, Saint Louis, MO, USA) to minimize auto‐oxidation of the spin probe. Three‐millimeter aortic rings were washed once in PSS and again in modified Krebs–HEPES buffer. Rings were then incubated for 60 min at 37 °C in 200 μL Krebs‐–HEPES buffer containing 0.5 mmol L^−1^ CMH and analyzed immediately on an MS300 X‐band EPR spectrometer (Magnettech, Berlin, Germany). Instrument settings were as follows: microwave frequency 9.83 Ghz, centerfield 3480 G, sweep 80 G, modulation amplitude 3.3 G, microwave power 40 mW, microwave attenuation 7, and receiver gain 30. A total of six sweeps were conducted lasting 8.7 s per sweep. The running average of the six sweeps was collected with the double integration (area under and over the baseline) of the triplet used to display the magnitude of the signal. The magnitude of this signal directly relates to the amount of superoxide that has been trapped by the CMH.

### Pulse‐wave velocity

To assess large artery stiffening, aortic PWV was measured as described previously (Henson *et al*., 2014). Briefly, mice were anesthetized under 2% isoflurane in a closed chamber anesthesia machine (V3000PK, Parkland Scientific, Coral Springs, FL) for ~1–3 min. Anesthesia was maintained via nose‐cone and mice were secured in a supine position on a heating board (~35 °C) to maintain body temperature. Velocities were measured with 4‐mm piezoelectric crystal, 20‐MHz Doppler probes (Indus Instruments, Webster, TX, USA) at the transverse aortic arch, and ~4‐cm distal at the abdominal aorta and collected using WinDAQ Pro+ software (DataQ Instruments, Akron, OH, USA). Absolute pulse arrival times were indicated by the sharp upstroke, or foot, of each velocity waveform analyzed with WinDAQ Waveform Browser (DataQ Instruments). Aortic pulse‐wave velocity was then calculated as the quotient of the separation distance, assessed to the nearest half millimeter by engineering caliper (typically ~4 cm) and difference in absolute arrival times.

### Western blots

Because both carotid arteries were used for functional measures and to provide adequate tissue for measurement of proteins, the thoracic aorta was excised and used for protein expression assays. Histological samples were saved as described below and the remaining aorta was cleared of perivascular adipose tissue while maintained in 4 °C PSS. Cleared aorta was then frozen in liquid nitrogen. Whole artery lysates were prepared as previously described (Durrant *et al*., 2009). Protein expression was assessed by standard Western blot procedures using primary antibodies against ribosomal S6K (S6K, 1:1000; 32 kDa; Cell Signaling, Danvers, MA, USA), phosphorylated S6K (p‐S6K, 1:1000; 32 kDa; Cell Signaling), eNOS (1:1000; 140 kDa; BD Transduction, San Jose, CA, SUA), ser1177 phosphorylated eNOS (ser1177 p‐eNOS, 1:1000; 140 kDa; Cell Signaling), nitrotyrosine (NT, 1:100; 25/55/160 kDa; Abcam, Cambridge, MA, USA), NOX4 (1:650; 67 kDa; Abcam), extracellular superoxide dismutase (ecSOD, 1:500; 31/35 kDa; Sigma), AMPK (1:500; 62 kDa; Cell Signaling), phosphorylated AMPK (Thr172 p‐AMPK, 1:1000; 62 kDa; Cell Signaling), PTEN (1:1000; 54 kDa; Cell Signaling), p27kip (1:1000; 27 kDa; Cell Signaling) and GAPDH (1:1000; 37 kDa; Cell Signaling), appropriate HRP‐conjugated secondary antibodies (Jackson Immunological, West Grove, PA, USA), and Supersignal ECL (Pierce, Rockford, IL, USA). Bands were visualized using a digital acquisition system (ChemiDoc‐It, UVP, Upland, CA, USA or Bio‐Rad ChemiDoc XRS+ with ImageLab Software) and quantified using ImageJ 1.42 software (NIH, Bethesda, MD, USA). To account for differences in protein loading, expression is normalized to GAPDH expression. Representative images are provided. The ratio of phosphorylated to total protein is calculated from the bands for a given sample lysate.

### Quantitative PCR

mRNA expression for *p19* and *Nox2* was measured in lysed aortas by qRT–PCR using the QuantiTect Reverse Transcription kit (Qiagen, Inc., Valencia, CA, USA) and FastStart SYBR Green Master (Roche Diagnostics Corporation, Roche Applied Science, Indianapolis, IN, USA) according to the manufacturer's protocols. Fold change in mRNA expression was calculated as the fold difference in expression of target mRNA to *18s* rRNA for each animal $2^{-(\mathrm{target}\;C_{T}\;-\;18s\;\;C_{T})}$; 18s rRNA QuantiTect Primer Assay: Qiagen, Inc.). *p19* mRNA primers: fwd‐CGCAGGTTCTTGGTCACTGT and rev‐TGTTCACGAAAGCCAGAGCG; *Nox2* mRNA primers: fwd‐TCCCAGAGAACACAGCATAAC and rev‐CTAGCCTGCTTATGGGATTCTT.

### Histology

Two‐millimeter rings of thoracic aorta with perivascular tissue intact were removed from the thoracic aorta directly distal to the greater curvature of the aortic arch and embedded in optimal cutting temperature (OCT) medium. Rings were sectioned (7 μm) and mounted on glass slides for histological analysis. Intima‐media area was measured on Maisson's trichrome (HT15, Sigma) stained sections of aorta using ImageJ. Collagen was quantified by picrosirius red stain as described previously (Donato *et al*., 2013; Henson *et al*., 2014), and green channel images from a RGB stack were utilized for densitometric quantification with ImageJ (NIH, Bethesda, MA, USA). Elastin was quantified by Verhoff's Van Geison stain as described previously (Donato *et al*., 2013; Henson *et al*., 2014), and 8‐bit gray‐scale images were utilized for densitometric quantification with ImageJ. AGEs were assessed by immunohistochemical visualization. Briefly, sections were washed and incubated in primary antibody (1:200, GeneTex 20055) or negative control (2.5% horse serum, Vector Labs) overnight, and AGEs were visualized using the appropriate secondary antibody and Vector Labs NovaRed (SK‐4800) Peroxidase substrate kit. Three separate, blinded observers scored images on a zero to three scale (0 = absence of appreciable positive stain, 1 = minimal positive stain, 2 = appreciable positive stain, 3 = highly positive stain). Scores for each section were averaged across observers and normalized to negative control sections. Calcium staining was performed on aorta sections by Von Kossa staining by the instructions provided by the kit manufacturer (Polysciences, Inc., Warrington, PA, USA), with staining in 3% silver nitrate for 40 min under UV light. A slide containing sections of bone was used as a positive control.

### Statistics

Repeated‐measures analysis of variance (ANOVA) was performed to assess differences in ACh and SNP dose responses as well as for GTT curves. One‐way ANOVAs were performed for all other analyses. Least‐squares differences post hoc tests were performed where appropriate. Data are presented as mean ± SEM. Significance was set at *P* < 0.05.

## Funding

This work was supported by awards from the National Institute on Aging; R01 AG040297, K02 AG045339, R21 AG04395, R37 AG013038, and KO1 AG046326, the National Heart, Lung, and Blood Institute R01 HL107120, and by the Merit Review Award 1I01BX002151 from the United States (US) Department of Veterans Affairs Biomedical Laboratory Research and Development Service. The contents do not represent the views of the US Department of Veterans Affairs or the United States Government.

## Conflict of interest

The authors have no conflict of interest to disclose.

## Author contributions

LA Lesniewski, AJ Donato, and DR Seals contributed to all aspects of the study, including the conception and design, data collection and analysis, and manuscript preparation. TJ LaRocca, AE Walker, BR Lawson, ML Zigler, MW Blimline, GC Bosshardt, DW Trott, and GD Henson contributed to the collection and analysis of the data and revision of the manuscript.

## Supporting information


**Fig. S1** Glucose tolerance in young and old untreated and rapamycin treated mice.
**Table S1** Total blood count and differential from old untreated and rapamycin (Rap) treated mice.

## References

[acel12524-bib-0001] Anisimov VN , Zabezhinski MA , Popovich IG , Piskunova TS , Semenchenko AV , Tyndyk ML , Yurova MN , Rosenfeld SV , Blagosklonny MV (2011) Rapamycin increases lifespan and inhibits spontaneous tumorigenesis in inbred female mice. Cell Cycle 10, 4230–4236. 22107964 10.4161/cc.10.24.18486

[acel12524-bib-0002] Avendano GF , Agarwal RK , Bashey RI , Lyons MM , Soni BJ , Jyothirmayi GN , Regan TJ (1999) Effects of glucose intolerance on myocardial function and collagen‐linked glycation. Diabetes 48, 1443–1447. 10389851 10.2337/diabetes.48.7.1443

[acel12524-bib-0003] Beisswenger PJ , Moore LL , Curphey TJ (1993) Relationship between glycemic control and collagen‐linked advanced glycosylation end products in type I diabetes. Diabetes Care 16, 689–694. 8495605 10.2337/diacare.16.5.689

[acel12524-bib-0004] Chen C , Liu Y , Liu Y , Zheng P (2009) mTOR regulation and therapeutic rejuvenation of aging hematopoietic stem cells. Sci. Signal. 2, ra75. 19934433 10.1126/scisignal.2000559 PMC4020596

[acel12524-bib-0005] D'Agostino RB , Vasan RS , Pencina MJ , Wolf PA , Cobain M , Massaro JM , Kannel WB (2008) General cardiovascular risk profile for use in primary care: the Framingham Heart Study. Circulation 117, 743–753. 18212285 10.1161/CIRCULATIONAHA.107.699579

[acel12524-bib-0006] Donato AJ , Eskurza I , Silver AE , Levy AS , Pierce GL , Gates PE , Seals DR (2007) Direct evidence of endothelial oxidative stress with aging in humans relation to impaired endothelium‐dependent dilation and upregulation of nuclear factor‐κB. Circ. Res. 100, 1659–1666. 17478731 10.1161/01.RES.0000269183.13937.e8

[acel12524-bib-0007] Donato AJ , Gano LB , Eskurza I , Silver AE , Gates PE , Jablonski K , Seals DR (2009) Vascular endothelial dysfunction with aging: endothelin‐1 and endothelial nitric oxide synthase. Am. J. Physiol. Heart Circ. Physiol. 297, H425–H432. 19465546 10.1152/ajpheart.00689.2008 PMC2711733

[acel12524-bib-0008] Donato AJ , Henson GD , Morgan RG , Enz RA , Walker AE , Lesniewski LA (2012) TNF‐α impairs endothelial function in adipose tissue resistance arteries of mice with diet‐induced obesity. Am. J. Physiol. Heart Circ. Physiol. 303, H672–H679. 22821989 10.1152/ajpheart.00271.2012 PMC3468456

[acel12524-bib-0009] Donato AJ , Walker AE , Magerko KA , Bramwell RC , Black AD , Henson GD , Lawson BR , Lesniewski LA , Seals DR (2013) Life‐long caloric restriction reduces oxidative stress and preserves nitric oxide bioavailability and function in arteries of old mice. Aging Cell 12, 772–783. 23714110 10.1111/acel.12103 PMC3772986

[acel12524-bib-0010] Durrant JR , Seals DR , Connell ML , Russell MJ , Lawson BR , Folian BJ , Donato AJ , Lesniewski LA (2009) Voluntary wheel running restores endothelial function in conduit arteries of old mice: direct evidence for reduced oxidative stress, increased superoxide dismutase activity and down‐regulation of NADPH oxidase. J. Physiol. 587, 3271–3285. 19417091 10.1113/jphysiol.2009.169771 PMC2727036

[acel12524-bib-0011] Eid AA , Ford BM , Bhandary B , de Cassia Cavaglieri R , Block K , Barnes JL , Gorin Y , Choudhury GG , Abboud HE (2013) Mammalian target of rapamycin regulates Nox4‐mediated podocyte depletion in diabetic renal injury. Diabetes 62, 2935–2947. 23557706 10.2337/db12-1504 PMC3717863

[acel12524-bib-0012] Fang Y , Westbrook R , Hill C , Boparai RK , Arum O , Spong A , Wang F , Javors MA , Chen J , Sun LY (2013) Duration of rapamycin treatment has differential effects on metabolism in mice. Cell Metab. 17, 456–462. 23473038 10.1016/j.cmet.2013.02.008 PMC3658445

[acel12524-bib-0013] Fleenor BS , Eng JS , Sindler AL , Pham BT , Kloor JD , Seals DR (2014) Superoxide signaling in perivascular adipose tissue promotes age‐related artery stiffness. Aging Cell 13, 576–578. 24341314 10.1111/acel.12196 PMC4326900

[acel12524-bib-0014] Flynn JM , O'Leary MN , Zambataro CA , Academia EC , Presley MP , Garrett BJ , Zykovich A , Mooney SD , Strong R , Rosen CJ , Kapahi P , Nelson MD , Kennedy BK , Melov S (2013) Late‐life rapamycin treatment reverses age‐related heart dysfunction. Aging Cell 12, 851–862. 23734717 10.1111/acel.12109 PMC4098908

[acel12524-bib-0015] Gano LB , Donato AJ , Pasha HM , Hearon CM Jr , Sindler AL , Seals DR (2014) The SIRT1 activator SRT1720 reverses vascular endothelial dysfunction, excessive superoxide production, and inflammation with aging in mice. Am. J. Physiol. Heart Circ. Physiol. 307, H1754–H1763. 25326534 10.1152/ajpheart.00377.2014 PMC4269699

[acel12524-bib-0016] Goldin A , Beckman JA , Schmidt AM , Creager MA (2006) Advanced glycation end products: sparking the development of diabetic vascular injury. Circulation 114, 597–605. 16894049 10.1161/CIRCULATIONAHA.106.621854

[acel12524-bib-0017] Greer EL , Dowlatshahi D , Banko MR , Villen J , Hoang K , Blanchard D , Gygi SP , Brunet A (2007) An AMPK‐FOXO pathway mediates longevity induced by a novel method of dietary restriction in *C. elegans* . Curr. Biol. 17, 1646–1656. 17900900 10.1016/j.cub.2007.08.047 PMC2185793

[acel12524-bib-0018] Gwinn DM , Shackelford DB , Egan DF , Mihaylova MM , Mery A , Vasquez DS , Turk BE , Shaw RJ (2008) AMPK phosphorylation of raptor mediates a metabolic checkpoint. Mol. Cell 30, 214–226. 18439900 10.1016/j.molcel.2008.03.003 PMC2674027

[acel12524-bib-0019] Harrison DE , Strong R , Sharp ZD , Nelson JF , Astle CM , Flurkey K , Nadon NL , Wilkinson JE , Frenkel K , Carter CS (2009) Rapamycin fed late in life extends lifespan in genetically heterogeneous mice. Nature 460, 392–395. 19587680 10.1038/nature08221 PMC2786175

[acel12524-bib-0020] Henson GD , Walker AE , Reihl KD , Donato AJ , Lesniewski LA (2014) Dichotomous mechanisms of aortic stiffening in high‐fat diet fed young and old B6D2F1 mice. Physiol. Rep. 2, 1–9. 10.1002/phy2.268 PMC4002248 24760522

[acel12524-bib-0021] Herbig U , Ferreira M , Condel L , Carey D , Sedivy JM (2006) Cellular senescence in aging primates. Science 311, 1257–1257. 16456035 10.1126/science.1122446

[acel12524-bib-0022] Howell JJ , Manning BD (2011) mTOR couples cellular nutrient sensing to organismal metabolic homeostasis. Trends Endocrinol. Metab. 22, 94–102. 21269838 10.1016/j.tem.2010.12.003 PMC3744367

[acel12524-bib-0023] Karunadharma PP , Basisty N , Dai D‐F , Chiao YA , Quarles EK , Hsieh EJ , Crispin D , Bielas JH , Ericson NG , Beyer RP , MacKay VL , MacCoss MJ , Rabinovitch PS (2015) Subacute calorie restriction and rapamycin discordantly alter mouse liver proteome homeostasis and reverse aging effects. Aging Cell 14, 547–557. 25807975 10.1111/acel.12317 PMC4531069

[acel12524-bib-0024] Lamming DW , Ye L , Katajisto P , Goncalves MD , Saitoh M , Stevens DM , Davis JG , Salmon AB , Richardson A , Ahima RS , Guertin DA , Sabatini DM , Baur JA (2012) Rapamycin‐induced insulin resistance is mediated by mTORC2 loss and uncoupled from longevity. Science 335, 1638–1643. 22461615 10.1126/science.1215135 PMC3324089

[acel12524-bib-0025] Lesniewski LA , Hosch SE , Neels JG , de Luca C , Pashmforoush M , Lumeng CN , Chiang S‐H , Scadeng M , Saltiel AR , Olefsky JM (2007) Bone marrow–specific Cap gene deletion protects against high‐fat diet–induced insulin resistance. Nat. Med. 13, 455–462. 17351624 10.1038/nm1550

[acel12524-bib-0026] Lesniewski LA , Zigler MC , Durrant JR , Donato AJ , Seals DR (2012) Sustained activation of AMPK ameliorates age‐associated vascular endothelial dysfunction via a nitric oxide‐independent mechanism. Mech. Ageing Dev. 133, 368–371. 22484146 10.1016/j.mad.2012.03.011 PMC3359767

[acel12524-bib-0027] Majumder S , Caccamo A , Medina DX , Benavides AD , Javors MA , Kraig E , Strong R , Richardson A , Oddo S (2012) Lifelong rapamycin administration ameliorates age‐dependent cognitive deficits by reducing IL‐1beta and enhancing NMDA signaling. Aging Cell 11, 326–335. 22212527 10.1111/j.1474-9726.2011.00791.x PMC3306461

[acel12524-bib-0028] Medvedik O , Lamming DW , Kim KD , Sinclair DA (2007) MSN2 and MSN4 link calorie restriction and TOR to sirtuin‐mediated lifespan extension in *Saccharomyces cerevisiae* . PLoS Biol. 5, e261. 17914901 10.1371/journal.pbio.0050261 PMC1994990

[acel12524-bib-0029] Mihaylova MM , Shaw RJ (2011) The AMPK signalling pathway coordinates cell growth, autophagy and metabolism. Nat. Cell Biol. 13, 1016–1023. 21892142 10.1038/ncb2329 PMC3249400

[acel12524-bib-0030] Miller RA , Harrison DE , Astle CM , Baur JA , Boyd AR , de Cabo R , Fernandez E , Flurkey K , Javors MA , Nelson JF , Orihuela CJ , Pletcher S , Sharp ZD , Sinclair D , Starnes JW , Wilkinson JE , Nadon NL , Strong R (2011) Rapamycin, but not resveratrol or simvastatin, extends life span of genetically heterogeneous mice. J. Gerontol. A Biol. Sci. Med. Sci. 66, 191–201. 20974732 10.1093/gerona/glq178 PMC3021372

[acel12524-bib-0031] Miller RA , Harrison DE , Astle CM , Fernandez E , Flurkey K , Han M , Javors MA , Li X , Nadon NL , Nelson JF , Pletcher S , Salmon AB , Sharp ZD , Van Roekel S , Winkleman L , Strong R (2014) Rapamycin‐mediated lifespan increase in mice is dose and sex dependent and metabolically distinct from dietary restriction. Aging Cell 13, 468–477. 24341993 10.1111/acel.12194 PMC4032600

[acel12524-bib-0032] Mitchell GF , Parise H , Benjamin EJ , Larson MG , Keyes MJ , Vita JA , Vasan RS , Levy D (2004) Changes in arterial stiffness and wave reflection with advancing age in healthy men and women the Framingham Heart Study. Hypertension 43, 1239–1245. 15123572 10.1161/01.HYP.0000128420.01881.aa

[acel12524-bib-0033] Mitchell GF , Hwang S‐J , Vasan RS , Larson MG , Pencina MJ , Hamburg NM , Vita JA , Levy D , Benjamin EJ (2010) Arterial stiffness and cardiovascular events the Framingham Heart Study. Circulation 121, 505–511. 20083680 10.1161/CIRCULATIONAHA.109.886655 PMC2836717

[acel12524-bib-0034] Mozaffarian D , Benjamin EJ , Go AS , Arnett DK , Blaha MJ , Cushman M , de Ferranti S , Després J‐P , Fullerton HJ , Howard VJ , Huffman MD , Judd SE , Kissela BM , Lackland DT , Lichtman JH , Lisabeth LD , Liu S , Mackey RH , Matchar DB , McGuire DK , Mohler ER , Moy CS , Muntner P , Mussolino ME , Nasir K , Neumar RW , Nichol G , Palaniappan L , Pandey DK , Reeves MJ , Rodriguez CJ , Sorlie PD , Stein J , Towfighi A , Turan TN , Virani SS , Willey JZ , Woo D , Yeh RW , Turner MB (2015) Executive summary: heart disease and stroke statistics—2015 update: a report from the American Heart Association. Circulation 131, 434–441. 10.1161/CIR.0000000000000366 26811276

[acel12524-bib-0035] Murakami H , Murakami R , Kambe F , Cao X , Takahashi R , Asai T , Hirai T , Numaguchi Y , Okumura K , Seo H (2006) Fenofibrate activates AMPK and increases eNOS phosphorylation in HUVEC. Biochem. Biophys. Res. Commun. 341, 973–978. 16442496 10.1016/j.bbrc.2006.01.052

[acel12524-bib-0036] Neff F , Flores‐Dominguez D , Ryan DP , Horsch M , Schroder S , Adler T , Afonso LC , Aguilar‐Pimentel JA , Becker L , Garrett L , Hans W , Hettich MM , Holtmeier R , Holter SM , Moreth K , Prehn C , Puk O , Racz I , Rathkolb B , Rozman J , Naton B , Ordemann R , Adamski J , Beckers J , Bekeredjian R , Busch DH , Ehninger G , Graw J , Hofler H , Klingenspor M , Klopstock T , Ollert M , Stypmann J , Wolf E , Wurst W , Zimmer A , Fuchs H , Gailus‐Durner V , Hrabe de Angelis M , Ehninger D (2013) Rapamycin extends murine lifespan but has limited effects on aging. J. Clin. Invest. 123, 3272–3291. 23863708 10.1172/JCI67674 PMC3726163

[acel12524-bib-0037] Pestell RG , Albanese C , Reutens AT , Segall JE , Lee RJ , Arnold A (1999) The cyclins and cyclin‐dependent kinase inhibitors in hormonal regulation of proliferation and differentiation 1. Endocr. Rev. 20, 501–534. 10453356 10.1210/edrv.20.4.0373

[acel12524-bib-0038] Rippe C , Lesniewski L , Connell M , LaRocca T , Donato A , Seals D (2010) Short‐term calorie restriction reverses vascular endothelial dysfunction in old mice by increasing nitric oxide and reducing oxidative stress. Aging Cell 9, 304–312. 20121721 10.1111/j.1474-9726.2010.00557.x PMC2894368

[acel12524-bib-0039] Rossi R , Nuzzo A , Origliani G , Modena MG (2008) Prognostic role of flow‐mediated dilation and cardiac risk factors in post‐menopausal women. J. Am. Coll. Cardiol. 51, 997–1002. 18325438 10.1016/j.jacc.2007.11.044

[acel12524-bib-0040] Seals D , Jablonski K , Donato A (2011) Aging and vascular endothelial function in humans. Clin. Sci. 120, 357–375. 21244363 10.1042/CS20100476 PMC3482987

[acel12524-bib-0041] Sun C , Zhao J , Jin Y , Hou C , Zong W , Lu T , Li H , Gao J (2014) PTEN regulation of the proliferation and differentiation of auditory progenitors through the PTEN/PI3K/Akt‐signaling pathway in mice. NeuroReport 25, 177. 24481416 10.1097/WNR.0000000000000069 PMC3906289

[acel12524-bib-0042] Wilkinson JE , Burmeister L , Brooks SV , Chan CC , Friedline S , Harrison DE , Hejtmancik JF , Nadon N , Strong R , Wood LK , Woodward MA , Miller RA (2012) Rapamycin slows aging in mice. Aging Cell 11, 675–682. 22587563 10.1111/j.1474-9726.2012.00832.x PMC3434687

[acel12524-bib-0043] Yeboah J , Crouse JR , Hsu F‐C , Burke GL , Herrington DM (2007) Brachial flow‐mediated dilation predicts incident cardiovascular events in older adults the cardiovascular health study. Circulation 115, 2390–2397. 17452608 10.1161/CIRCULATIONAHA.106.678276

[acel12524-bib-0044] Zakikhani M , Blouin M‐J , Piura E , Pollak MN (2010) Metformin and rapamycin have distinct effects on the AKT pathway and proliferation in breast cancer cells. Breast Cancer Res. Treat. 123, 271–279. 20135346 10.1007/s10549-010-0763-9

