# Supplementary material for: Dietary rapamycin supplementation reverses age‐related vascular dysfunction and oxidative stress, while modulating nutrient‐sensing, cell cycle, and senescence pathways
Source: Aging Cell. 2016 Sep 22;16(1):17–26. doi: 10.1111/acel.12524 (PMC5242306; doi:10.1111/acel.12524)
Supplement: Supplementary file 1 — Fig. S1 Glucose tolerance in young and old untreated and rapamycin treated mice. Table S1 Total blood count and differential from old untreated and rapamycin (Rap) treated mice. [file ACEL-16-17-s001.docx]

**SUPPORTING INFORMATION**

**Supplemental Table.** Total blood count and differential from old untreated and rapamycin (Rap) treated mice.

|  | | Old | Old Rap |
| --- | --- | --- | --- |
| White Blood Cells (units) | | 9.5 ± 1.2 | 9.0 ± 0.8 |
| Hemoglobin (units) | | 15.3 ± 0.9 | 16.0 ± 0.5 |
| Hematocrit (units) | | 48.8 ± 2.6 | 50.9 ± 1.7 |
| Platelets (units) | | 1366 ± 69 | 1213 ± 64 |
| Neutrophils | **%** | 11.1 ± 1.3 | 11.4 ± 1.3 |
|  | **# (10^9^ cell/L)** | 1.0 ± 0.1 | 1.0 ± 0.2 |
| Lymphocytes | **%** | 84.8 ± 1.4 | 84.2 ± 1.6 |
|  | **# (10^9^ cell/L)** | 8.1 ± 1.1 | 7.6 ± 0.6 |
| Monocytes | **%** | 3.2 ± 0.3 | 3.1 ± 0.4 |
|  | **# (10^9^ cell/L)** | 0.30 ± 0.03 | 0.28 ± 0.06 |
| Eosinophils | **%** | 0.2 ± 0.1 | 0.4 ± 0.2 |
|  | **# (10^9^ cell/L)** | 0.02 ± 0.01 | 0.04 ± 0.02 |
| Basophils | **%** | 0.8 ± 0.1 | 0.9 ± 0.3 |
|  | **# (10^9^ cell/L)** | 0.07 ± 0.02 | 0.09 ± 0.03 |

**Supplemental Figure Legends**

**Supplemental Figure 1. Glucose tolerance in young and old untreated and rapamycin treated mice.** (**A**) Glucose tolerance assessed by measuring blood glucose concentrations at time 0, 15, 30, 45, 60 and 90 min after injection of glucose (2 g/mg, ip) in fasted (2 hr) young (N=8) and old (N=12) mice before (Pre) and after 6 weeks of dietary rapamycin treatment (Post). Differences in glucose tolerance assessed by Repeated Measures ANOVA and differences at individual time points were assessed by One-Way ANOVA with LSD post hoc where appropriate. * Denotes difference from untreated Young, † Denotes difference from untreated Old. Data are means±SEM, P≤0.05

Young NC

Old NC

Y Rap

O Rap

**Supplemental Figure 1**

**A**

†

†

†

†

*

*
